# Palliative radiotherapy in symptomatic locally advanced gastric cancer: A phase II trial

**DOI:** 10.1002/cam4.2021

**Published:** 2019-02-20

**Authors:** Jeremy Tey, Huili Zheng, Yu Y. Soon, Cheng N. Leong, Wee Y. Koh, Keith Lim, Jimmy B. Y. So, Asim Shabbir, Ivan W. K. Tham, Jiade Lu

**Affiliations:** ^1^ Department of Radiation Oncology National University Hospital National Cancer Institute of Singapore Singapore Singapore; ^2^ National Registry of Diseases Singapore Singapore; ^3^ Department of Surgery National University Hospital Singapore Singapore; ^4^ Shanghai Heavy ion and Proton Centre Shanghai China

**Keywords:** bleeding, gastric cancer, palliative, radiotherapy

## Abstract

To evaluate the response and quality of life of palliative gastric radiotherapy in patients with symptomatic locally advanced gastric cancer. Patients with bleeding, pain or obstruction and were treated with palliative gastric radiotherapy to a dose of 36 Gy in 12 daily fractions. The primary outcomes were symptom response rates. Secondary outcomes included overall survival, adverse events and proportion of patients with ≥10‐point absolute improvement in the fatigue, nausea/vomiting and pain subscales in the EORTC Qualify of Life Questionnaire C30 (EORTC QLQ‐C30) and dysphagia/pain subscales in the gastric specific module (STO22) at the end of RT and 1 month after the completion of radiotherapy. Fifty patients were accrued. Median survival duration was 85 days. 40/50 patients (80%) with bleeding, 2/2 (100%) patients with obstruction and 1/1 (100%) patient with pain responded to radiotherapy. Improvements fatigue, nausea/vomiting and pain subscales of the EORTC QLQ‐C30 was seen in 50%, 28% and 44% of patients at the end of RT and in 63%, 31% and 50% of patients 1 month after RT. Improvements in dysphagia/pain subscales of the STO22 was seen in 42% and 28% of patients at then end of RT and 44% and 19% of patients 1 month after RT. Two patients (5%) had grade 3 anorexia and gastritis. Palliative gastric radiotherapy was effective, well tolerated and resulted in improvement in fatigue, dysphagia and pain at the end of radiotherapy and 1 month after the completion of radiotherapy in a significant proportion of patients.

## 1 INTRODUCTION

Gastric cancer is the fifth most common malignancy in the world, and is the third leading causing of global mortality.^1^ Local tumor progression is a cause of presenting symptoms in many patients with recurrent or locally advanced gastric cancers. This can include bleeding (melena, hematemesis), loss of appetite, gastric pain, or obstruction (post prandial fullness, dysphagia, nausea or vomiting).

Different treatments have been employed to treat symptoms from local tumor extension. These include argon plasma laser coagulation,^2^ palliative gastrectomy,^3^, ^4^, ^5^, ^6^, ^7^ gastric bypass,^8^, ^9^ stenting,^10^ palliative chemotherapy^11^ and palliative radiotherapy (RT).^12^, ^13^, ^14^, ^15^, ^16^ Palliative RT is attractive as it is non‐invasive and has been shown to be effective in randomized trials in palliating bleeding and obstructive symptoms in patients with advanced lung^17^ and bladder cancers.^18^

However, there is limited research on palliation of symptoms resulting from primary gastric tumor. A systematic review and meta‐analysis of published studies on the efficacy of palliative gastric RT found that there were no prospective studies examining this issue.^19^ The interpretation of the retrospective studies on the efficacy of palliative gastric RT is limited by the use of concurrent chemotherapy, different definitions of endpoints and the use of different radiation dose fractionation schedules. In addition, there was no assessment of patient reported outcomes or quality of life.

Therefore, the aim of this single arm phase II trial was to evaluate the symptom response and quality of life in patients with symptomatic locally advanced gastric cancer treated with palliative gastric RT.

## 2 METHODS

This trial was approved by the National Healthcare Group Institutional Review Board and registered with Clinicaltrials.gov (NCT01341756).

### 2.1 Eligibility

Eligible patients had biopsy proven adenocarcinoma of the stomach treated with palliative intent. Patients must present with gastric bleeding as the index symptom. They must not have had prior abdominal RT and not have concurrent chemotherapy. Patients younger than 21 years old were excluded. All subjects must have given written informed consent prior to simulation.

### 2.2 Radiotherapy

The prescription dose was 36 Gy in 12 daily fractions, 5 days a week, over two and a half weeks. This dose was selected as a study from MD Anderson suggested that low biologically effective dose (BED) regimens (<41 Gy) predicted for poorer local control compared to higher BED regimens.^20^ This dose fractionation equates to a BED of 48.6 Gy, which is more than 41 Gy and is just 2 more fractions from the commonly employed schedule of 30 Gy in 10 fractions.

#### 2.2.1 Computed tomography (CT) simulation

Patients were simulated in the supine position with arms up. The CT scan was performed without contrast with 5 mm thick slices.

#### 2.2.2 Target volume definition

The gross tumor volume (GTV) was the whole stomach. The partial stomach was treated if the clinician was able to localise the tumor on CT scan. The clinical target volume (CTV) included the GTV plus a 0.5 cm margin as appropriate to account for microscopic tumor extension. The planning target volume (PTV) included the CTV plus a 1 cm margin.

#### 2.2.3 Planning goals

Multi‐field conformal photon beams with multi‐leaf collimation (MLC) were used to spare the normal critical structures such as the kidneys and liver. The most common beam arrangement was an anterior‐posterior field with parallel opposed lateral fields. Normalization of the treatment plan to cover 95% of the PTV with the prescription dose was performed. The minimum PTV dose must not fall below 95% of the prescription dose.

#### 2.2.4 Dose constraints

The volume of combined kidneys receiving 15 Gy or more (*V*_15_) should be ≤50%. The volume of liver receiving 27 Gy or more (*V*_27_) should be ≤60%. The maximum dose (*D*_max_) of spinal cord should be ≤36 Gy.

### 2.3 Primary endpoints

#### 2.3.1 Bleeding

A response to RT was defined as no further blood transfusion needed at the completion of RT and/or no further melena episode at the completion of RT. We hypothesized that a course of RT with dose fractionation of 36 Gy in 12 fractions, given daily (Monday to Friday) 3 Gy per fraction (BED >41 Gy) increases the response rate of bleeding from 55% (historical)^12^ to 75%.

#### 2.3.2 Pain

A partial response to RT was scored when the patient had decreased pain or same pain but decreased analgesia. A complete response was scored if their pain resolved post treatment.

#### 2.3.3 Obstruction

Obstruction was defined in three categories in decreasing severity: patients requiring parenteral nutrition, patients tolerating liquids and patients tolerating solids. An improvement upward of one category was quantified as a partial response. Resolution of obstructive symptoms was scored as a complete response.

### 2.4 Secondary endpoints

#### 2.4.1 Duration of symptom response

The duration of response is defined as the time from response in patients who achieved symptom palliation until symptom recurrence or death.

#### 2.4.2 Percent net symptom relief

The duration of relief from bleeding/pain/obstruction and survival from the completion of RT was determined for each patient. Multiplying the ratio of the duration of relief from bleeding/pain/obstruction and survival time by 100 yielded the “percent net symptom relief”. This represented the percentage of the remaining patient's life after RT that was spent with relief of index symptom and without need for further treatment.^21^

#### 2.4.3 Overall survival

Overall survival referred to the time from the completion of RT to death from all causes.

#### 2.4.4 Health related quality of life (HRQOL)

The European Organization for Research and Treatment of Cancer Qualify of Life Questionnaire C30 (EORTC QLQ‐C30) is designed to assess the quality of life of cancer patients. It comprises both multi‐item and single item measures. These include: five functional subscales (physical, role, emotional, cognitive, social), three symptom subscales (fatigue, nausea/vomiting, pain), a global health status/HRQOL subscale and six single items (dyspnea, insomnia, appetite loss, constipation, diarrhea, financial problems). All subscales and items range from 0 to 100. A high score on the functional and global health status subscales represents high response level, while a high score on the symptom subscales and single items represents high symptom level.^22^

The gastric specific module (STO22) was used on patients with gastric cancer and this complements the EORTC QLQ‐C30. It consists of one functional subscale (body image), five symptom subscales (dysphagia, eating restrictions, pain, reflux, anxiety) and three single items (dry mouth, taste problem, hair loss). Scoring was performed in accordance to the EORTC scoring manual. A high score on the functional subscale represents high response level, while a high score on the symptom subscales and single items represents greater level of symptoms.^23^

HRQOL assessments using the EORTC QLQ‐C30 and STO22 were performed at baseline, at the end of RT and at 1 month after the completion of RT for each patient. A change in score of ≥10 points was considered to be clinically significant, a priori. Domains of interest include fatigue, nausea/vomiting and pain subscales in the EORTC QLQ‐C30, and dysphagia and pain subscales in the STO22 at the end of RT and 1 month after the completion of RT. For pain, the hypothesis is that RT will result in ≥30% of the patients having a ≥10‐point absolute improvement in the pain subscales of both the EORTC QLQ‐C30 and STO22 at the end of RT and 1 month after the completion of RT. For obstruction, the hypothesis is that RT will result in ≥30% of the patients having a ≥10‐point absolute improvement in the nausea/vomiting subscales of the EORTC QLQ‐C30 and dysphagia subscales of the STO22 at the end of RT and 1 month after the completion of RT.

#### 2.4.5 Treatment toxicity

Treatment toxicity was scored using the Common Toxicity Criteria for Adverse Events (CTCAE) v3.0.^24^ Assessments were done weekly during RT.

### 2.5 Follow up

All patients were followed up at 1 month after the completion of RT treatment and thereafter at 3 monthly intervals with physical examination and complete blood cell counts.

### 2.6 Statistical analysis

#### 2.6.1 Determination of sample size

This was a single arm phase 2 trial conducted at an academic instutition. The sample size was calculated with one‐sided significance level of 95% and statistical power of 95% with dichotomous outcome. We hypothesized that the bleeding symptom response rate in this trial would be 75% compared to the historical value of 55%.^12^ Factoring in an assumed drop‐out rate of 5%, a sample size of at least 63 patients must be recruited to achieve the desired statistical power.

#### 2.6.2 Statistical analyses

For the primary endpoints, the proportions of patients experiencing symptom relief were calculated. Univariable and multivariable logistic regressions were performed to examine the effects of age, gender, Eastern Cooperative Oncology Group (ECOG) performance status, TNM stage classification and timing of chemotherapy on bleeding response. For the secondary endpoints, the median duration of symptom response, mean percent net symptom relief and median survival duration were calculated where applicable. Univariable and multivariable cox proportional hazard regressions were performed to determine the effects of the above factors and bleeding response on overall survival. For HRQOL measures, the proportion of patients who achieved ≥10 points improvement from baseline in the various HRQOL domains at the end of RT and 1 month after the completion of RT were calculated. For all analyses, two‐sided *P* values of less than 0.05 were considered statistically significant. All analyses were done using STATA SE 13.

## 3 RESULTS

**Figure 1:**
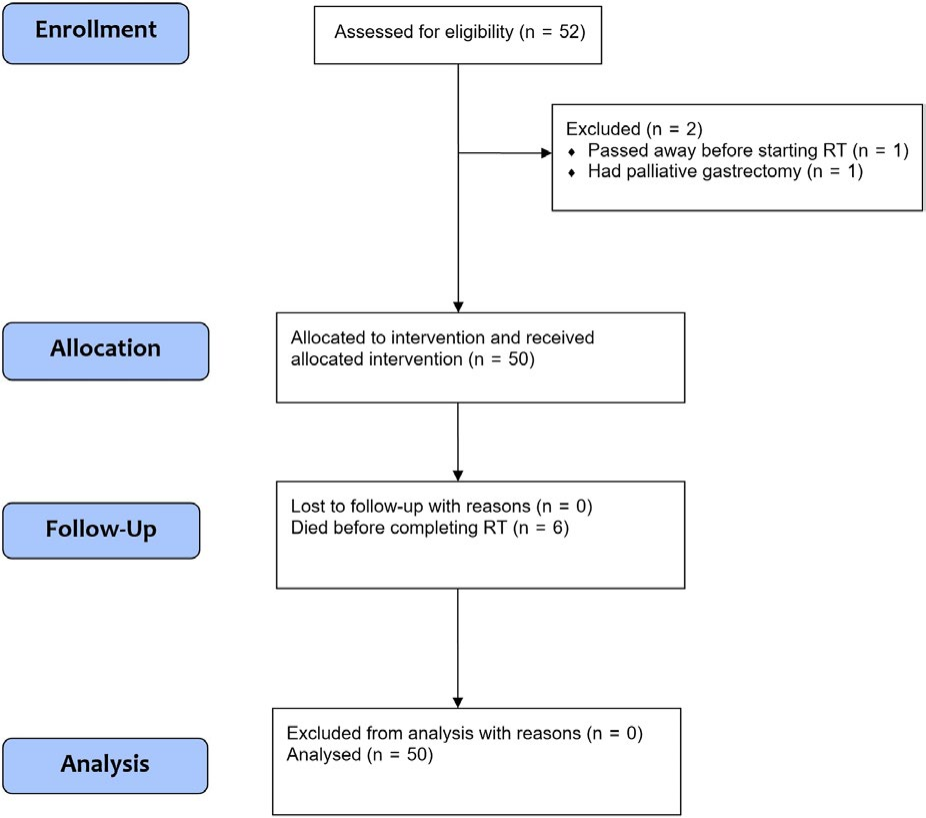
Consort flow diagram

From October 2009 to July 2014, 52 patients were enrolled (Figure 1). One patient had palliative gastrectomy and one patient passed away before starting treatment. The remaining 50 patients with symptomatic locally advanced/recurrent gastric cancer were managed with palliative intent using RT alone. All patients were CT planned. All patients presented with gastric bleeding as the index symptom, two patients had gastric pain and one patient had gastric obstruction concurrently. Six patients passed away from non‐treatment related causes before completing treatment and the remaining 44 patients completed protocol treatment. Among the 50 patients included in this study, the median age was 75 years (range: 23‐92). 37 (74%) patients had known distant metastatic disease at the time of treatment (Table 1).

**Table 1:** Patient characteristics (n = 50)

	N	%
Gender		
Male	29	58
Female	21	42
Age		
<60	5	10
60‐69	10	20
70‐79	16	32
80‐89	13	26
≥90	6	12
ECOG performance status		
1	21	42
2	24	48
3	4	8
4	1	2
T stage		
<T4	32	64
T4	18	36
Disease status		
Locally advanced (M0)	13	26
Metastatic (M1)	37	74
Nodal positivity		
No (N0)	9	18
Yes (N1)	41	82
Chemotherapy		
None	38	76
Before RT	5	10
After RT	7	14

ECOG, Eastern Cooperative Oncology Group; RT, radiotherapy.

### 3.1 Treatment response

#### 3.1.1 Relief from bleeding

40/50 patients (80%) with bleeding responded to RT. Among the patients who responded to RT, the median duration of response was 102 days (range: 2‐1031) and the mean percent net relief was 95%. Out of the 10 patients who did not respond to RT, six patients died before completing treatment. Their deaths were attributed to disease progression and not treatment toxicity. These 10 patients were scored as a non‐response to RT. Both univariable and multivariable analysis showed that age, gender, ECOG performance status, TNM stages and timing of chemotherapy were not associated with bleeding response (Table 2).

**Table 2:** Univariable and multivariable logistic regression of factors associated with symptom response (n = 50)

	Univariable		Multivariable	
	OR (95% CI)	*P* value	OR (95% CI)	*P* value
Age				
<60	1.00		1.00	
60‐69	0.58 (0.04‐7.66)	0.682	0.17 (0.002‐15.90)	0.448
70‐79	0.55 (0.05‐6.27)	0.630	0.43 (0.01‐22.83)	0.680
80‐89	3.00 (0.15‐59.89)	0.472	4.55 (0.06‐369.69)	0.499
>=90	NA	NA	NA	NA
Gender				
Male	1.00		1.00	
Female	1.11 (0.27‐4.55)	0.886	0.32 (0.03‐3.81)	0.370
ECOG performance status				
1	1.00		1.00	
2	0.40 (0.07‐2.32)	0.307	0.52 (0.06‐4.91)	0.571
3	0.11 (0.01‐1.21)	0.071	0.03 (0.0003‐1.99)	0.099
4	NA	NA	NA	NA
T stage				
<T4	1.00		1.00	
T4	0.48 (0.12‐1.96)	0.308	1.22 (0.10‐14.90)	0.875
Disease status				
Locally advanced (M0)	1.00		1.00	
Metastatic (M1)	0.66 (0.12‐3.60)	0.630	1.28 (0.09‐17.55)	0.851
Nodal positivity				
No (N0)	1.00		1.00	
Yes (N1)	1.18 (0.20‐6.79)	0.854	1.37 (0.11‐16.57)	0.803
Chemotherapy				
None	1.00		1.00	
Before RT	0.40 (0.06‐2.82)	0.358	1.93 (0.08‐44.15)	0.681
After RT	NA	NA	NA	NA

OR was not available for some groups as all patients in those groups either died or survived.

ECOG, Eastern Cooperative Oncology Group; OR, odds ratio; CI, confidence interval; RT, radiotherapy; NA, not applicable.

#### 3.1.2 Relief from pain

1/1 patient (100%) with pain responded to RT. The duration of response was 121 days for this patient and he had reduced analgia requirements post RT. The mean percent net relief was 100%.

#### 3.1.3 Relief from obstruction

2/2 (100%) patients with obstruction responded to RT. The duration of response was 27 days and 428 days for these patients. Both patients had dysphagia to solids that resolved at the end of RT. The mean percent net relief was 100% for both patients.

### 3.2 Survival

Thirty six of fifty (72%) patients were alive at 1 month after the completion of RT. The median survival duration was 83 days (range: 2‐1225). Both univariable and multivariable analysis showed that the risk of death was higher for patients with metastatic disease, HR 3.7 (95% CI 1.05‐13.07), *P* = 0.04, and those who did not respond to RT for bleeding, HR 0.20 (95% CI 0.07‐0.57), *P* < 0.01 (Table 3).

**Table 3:** Univariable and multivariable cox regression of factors associated with death from all causes (n = 50)

	Univariable		Multivariable	
	HR (95% CI)	*P* value	HR (95% CI)	*P* value
Age				
<60	1.00		1.00	
60‐69	1.65 (0.56‐4.86)	0.367	1.06 (0.29‐3.91)	0.934
70‐79	1.34 (0.48‐3.71)	0.573	1.05 (0.32‐3.46)	0.942
80‐89	0.47 (0.16‐1.43)	0.185	0.46 (0.12‐1.83)	0.272
>=90	0.58 (0.17‐1.97)	0.386	1.19 (0.20‐7.07)	0.850
Gender				
Male	1.00		1.00	
Female	0.72 (0.40‐1.30)	0.278	0.63 (0.31‐1.26)	0.189
ECOG				
1	1.00		1.00	
2	1.29 (0.71‐2.36)	0.405	1.67 (0.86‐3.25)	0.131
3	2.61 (0.87‐7.84)	0.089	1.32 (0.26‐6.67)	0.738
4	15.24 (1.64‐141.35)	0.017	4.74 (0.42‐53.61)	0.208
T stage				
<T4	1.00		1.00	
T4	1.27 (0.69‐2.33)	0.441	0.94 (0.43‐2.03)	0.867
Disease status				
Locally advanced (M0)	1.00		1.00	
Metastatic (M1)	2.32 (1.14‐4.75)	0.021	3.70 (1.05‐13.07)	0.042
Nodal positivity				
No (N0)	1.00		1.00	
Yes (N1)	0.92 (0.44‐1.92)	0.830	0.49 (0.19‐1.27)	0.141
Chemotherapy				
None	1.00		1.00	
Before RT	3.82 (1.38‐10.54)	0.010	1.88 (0.41‐8.69)	0.420
After RT	0.87 (0.38‐1.98)	0.737	0.76 (0.25‐2.36)	0.641
Bleeding response to RT				
No	1.00		1.00	
Yes	0.20 (0.09‐0.44)	<0.001	0.20 (0.07‐0.57)	0.003

ECOG, Eastern Cooperative Oncology Group; HR, hazard ratio; CI, confidence interval; RT, radiotherapy.

### 3.3 HRQOL

The completion rates for both the EORTC QLQ‐C30 and STO22 were 98% (49/50) at baseline, 80% (36/45) at the end of RT and 42% (16/38) at 1 month after the completion of RT.

**Figure 2:**
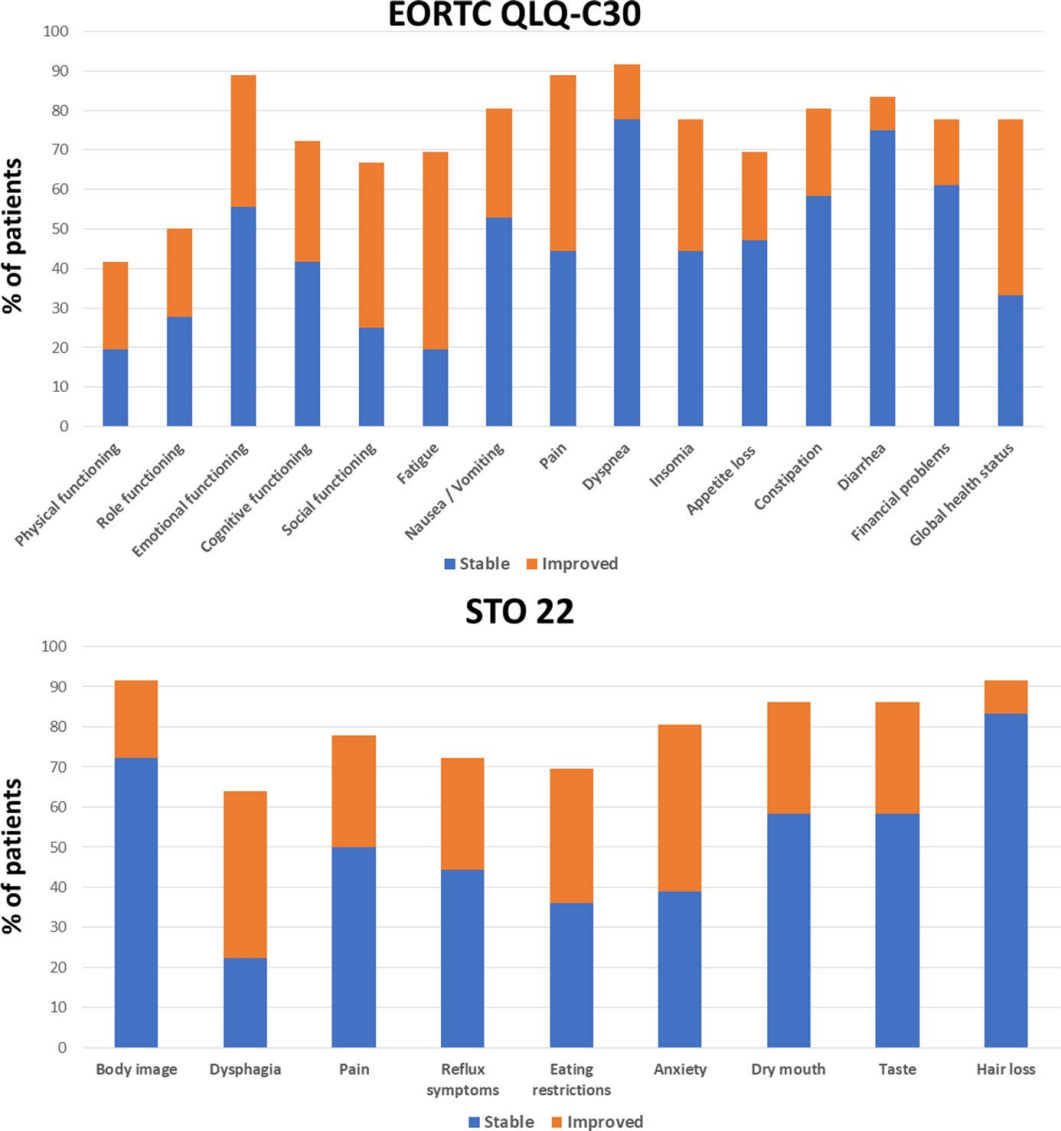
Proportion of patients with clinically significant improvement or stability in the various HRQOL domains from baseline to the end of RT

At the end of RT, 50%, 28% and 44% of the patients achieved improvements in fatigue, nausea/vomiting and pain subscales of the EORTC QLQ‐C30 respectively (Table 4). Significant improvements were also seen in emotional/cognitive/social functioning, insomnia and global health status subscales of the EORTC QLQ‐C30. 42% and 28% of the patients achieved improvements in dysphagia and pain subscales of the STO22. Significant improvements were also seen in eating restrictions and anxiety subscales. Figure 2 shows the proportion of patients with clinically significant improvement or stability in the various HRQOL domains from baseline to the end of RT.

**Table 4:** EORTC QLQ‐C30 and STO22 scores and proportion of patients with improvement at the end of RT (n = 36)

	Baseline	End of RT	Patients with improvement, n (%)
	Mean (SD)	Mean (SD)	
EORTC QLQ‐C30			
Physical functioning^a^	55.9 (27.5)	50.0 (31.8)	8 (22.2)
Role functioning^a^	54.2 (34.6)	50.0 (36.1)	9 (25.0)
Emotional functioning^a^	81.7 (19.7)	86.8 (21.9)	12 (33.3)
Cognitive functioning^a^	85.6 (17.9)	83.8 (22.0)	11 (30.6)
Social functioning^a^	75.0 (22.7)	75.0 (29.1)	15 (41.7)
*Fatigue* b	*43.2 (24.2)*	*35.8 (29.0)*	*18 (50.0)*
*Nausea/vomiting* b	*17.1 (24.4)*	*15.7 (22.5)*	*10 (27.8)*
*Pain* b	*26.9 (30.2)*	*15.7 (26.1)*	*16 (44.4)*
Dyspnea^b^	10.2 (25.0)	5.6 (18.7)	5 (13.9)
Insomnia^b^	28.7 (33.0)	24.1 (31.5)	12 (33.3)
Appetite loss^b^	38.0 (33.9)	42.6 (34.4)	8 (22.2)
Constipation^b^	18.5 (27.0)	14.8 (23.2)	8 (22.2)
Diarrhea^b^	5.6 (14.9)	10.2 (23.7)	3 (8.3)
Financial problems^b^	20.4 (30.1)	22.2 (30.9)	6 (16.7)
Global health status/quality of life^a^	49.3 (25.8)	55.1 (20.2)	16 (44.4)
STO22			
Body image^a^	87.0 (25.5)	91.7 (24.4)	7 (19.4)
*Dysphagia* b	*21.0 (20.7)*	*16.4 (18.1)*	*15 (41.7)*
*Pain* b	*18.3 (25.0)*	*14.4 (24.1)*	*10 (27.8)*
Reflux symptoms^b^	16.0 (23.8)	11.7 (21.4)	10 (27.8)
Eating restrictions^b^	28.5 (23.0)	23.1 (25.8)	12 (33.3)
Anxiety^b^	22.8 (28.7)	15.7 (27.0)	15 (41.7)
Dry mouth^b^	24.1 (27.2)	18.5 (24.5)	10 (27.8)
Taste problem^b^	22.2 (30.9)	16.7 (29.3)	10 (27.8)
Hair loss^b^	8.6 (28.4)	3.8 (10.8)	3 (8.6)

Domains of interest are italicized.

SD, standard deviation.

a A high score represents high response level.

b A high score represents high symptom level.

**Figure 3:**
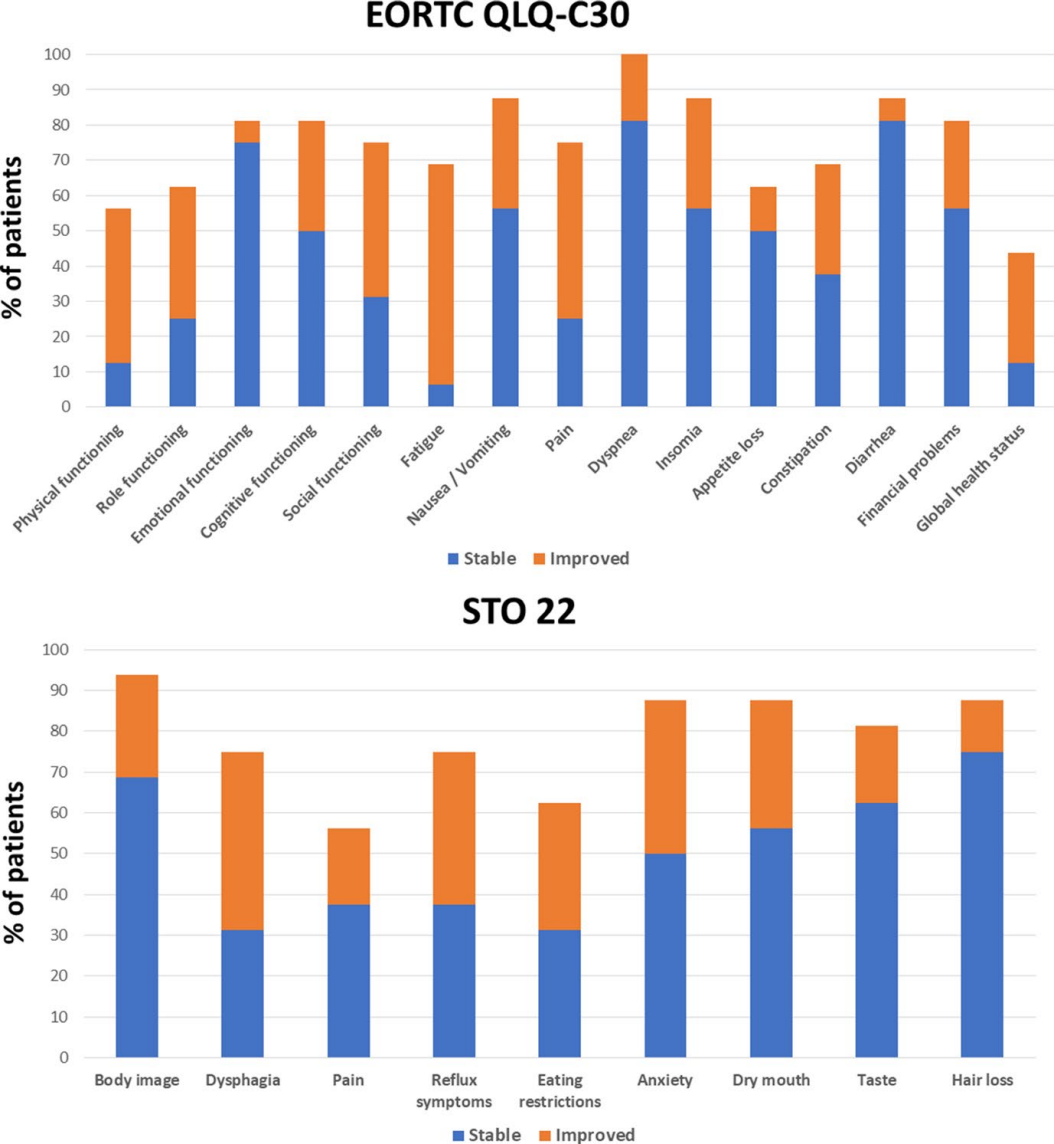
Proportion of patients with clinically significant improvement or stability in the various HRQOL domains from baseline to 1 month after the completion of RT

At 1 month after the completion of RT, 63%, 31% and 50% of the patients achieved improvements in fatigue, nausea/vomiting and pain subscales of the EORTC QLQ‐C30 respectively (Table 5). Significant improvements were also seen in physical/role/cognitive/social functioning, insomnia, constipation and global health status subscales. 44% and 19% of the patients achieved improvements in dysphagia and pain subscales of the STO22. Substantial improvements were also seen in reflux symptoms, eating restrictions, anxiety and dry mouth subscales. Figure 3 shows the proportion of patients with clinically significant improvement or stability in the various HRQOL domains from baseline to 1 month after the completion of RT.

**Table 5:** EORTC QLQ‐C30 and STO22 scores and proportion of patients with improvement at 1 month after the completion of RT (n = 16)

	Baseline	1 month after the completion of RT	Patients with improvement, n (%)
	Mean (SD)	Mean (SD)	
EORTC QLQ‐C30			
Physical functioning^a^	66.7 (23.0)	65.8 (28.7)	7 (43.8)
Role functioning^a^	66.7 (29.8)	65.6 (37.7)	6 (37.5)
Emotional functioning^a^	87.0 (13.9)	82.8 (27.6)	1 (6.3)
Cognitive functioning^a^	91.7 (10.5)	92.7 (14.9)	5 (31.3)
Social functioning^a^	74.0 (21.9)	75.0 (32.2)	7 (43.8)
*Fatigue* a	*41.7 (20.1)*	*33.3 (27.8)*	*10 (62.5)*
*Nausea/vomiting* a	*15.6 (23.1)*	*9.4 (14.9)*	*5 (31.3)*
*Pain* a	*32.3 (32.5)*	*17.7 (27.5)*	*8 (50.0)*
Dyspnea^a^	10.4 (26.4)	0.0 (0.0)	3 (18.8)
Insomnia^a^	25 (37.5)	12.5 (24.0)	5 (31.3)
Appetite loss^a^	29.2 (31.9)	41.7 (31.0)	2 (12.5)
Constipation^a^	22.9 (33.8)	20.8 (20.6)	5 (31.3)
Diarrhea^a^	6.3 (18.1)	6.3 (13.4)	1 (6.3)
Financial problems^a^	27.1 (32.7)	20.8 (34.2)	4 (25.0)
Global health status/quality of life^a^	60.9 (18.2)	53.1 (26.0)	5 (31.3)
STO22			
Body image^a^	81.3 (32.1)	85.4 (34.4)	3 (18.8)
*Dysphagia* a	*22.2 (19.9)*	*18.8 (18.9)*	*7 (43.8)*
*Pain* a	*16.1 (23.9)*	*19.3 (27.8)*	*3 (18.8)*
Reflux symptoms^a^	15.3 (22.5)	10.4 (13.1)	6 (37.5)
Eating restrictions^a^	26.0 (23.5)	24.0 (28.2)	5 (31.3)
Anxiety^a^	25.7 (34.8)	16.0 (30.6)	6 (37.5)
Dry mouth^a^	20.8 (24.0)	18.8 (29.7)	5 (31.3)
Taste problem^a^	20.8 (29.5)	14.6 (24.3)	3 (18.8)
Hair loss^a^	14.3 (36.3)	8.9 (15.3)	2 (14.3)

Domains of interest are italicized.

SD, standard deviation.

A high score represents high response level.

a A high score represents high symptom level.

### 3.4 Toxicity

Grade 1 or 2 nausea was seen in 20 (40%) patients. Grade 1 or 2 fatigue was seen in six (12%) patients. Two patients (5%) developed grade 3 toxicities. Grade 3 gastritis was seen in one patient during the second week of treatment, requiring admission for 1 week of intravenous hydration, analgesia (panadeine) and proton pump inhibitors (omeprazole). Grade 3 anorexia was seen in one patient during the second week of treatment, which resolved spontaneously by the end of treatment.

## 4 DISCUSSION

This study showed that palliative gastric RT for bleeding, pain and obstruction was well tolerated and associated with high response rates. The response rates for bleeding, pain and obstruction were 80%, 100% and 100% respectively. The mean percent net relief showed that for patients with bleeding, pain and obstruction that responded to RT, the response lasted without the need for further treatment for 95%, 100% and 100% respectively, of their remaining life span. This was also reflected in the median duration of response of 102 days for bleeding, which was close to the median survival duration of 83 days. More than 30% of the patients reported significant improvements in the HRQOL domains of interest, such as fatigue and pain subscales of the EROTC QLQ‐C30 and dysphagia subscale of the STO22 at the end of RT. Significant improvements were also observed for the fatigue, nausea/vomiting and pain subscales of the EROTIC QLQ‐C30 and dysphagia subscale of the STO22 at 1 month after the completion of RT. Treatment was well tolerated with 5% of the patients having toxicities of grade 3 or higher.

All patients had bleeding as the index symptom. Concurrently, one patient had gastric pain and two patients had dysphagia to solids as the index symptoms. Although they responded to RT, we also noted that other patients had improvements in the nausea/vomiting, pain and dysphagia subscales in the EORTC QLQ‐C30 and STO22. Patients with mild pain and dysphagia may under‐report their symptoms to the treating physician as they may think that their symptoms are not severe. This highlights the importance of HRQOL measurements in studies of palliative interventions as it incorporates objective functioning and subjective wellbeing and allows for detection of meaningful differences in HRQOL before and after treatment. Approximately 50% of the patients had stable or improvement HRQOL at end of RT and at 1 month after the completion of RT, with improvement seen in approximately 25% of the patients. The improvements in domains of interest (fatigue, pain, nausea/vomiting subscales of the EORTaC QLQ‐C30 and dysphagia subscale of the STO22) were sustained from the end of RT to 1 month after the completion of RT.

Our results are consistent with studies on palliative RT for other sites. Duschesne et al reported 50%‐55% symptomatic improvement in hematuria for patients undergoing bladder RT.^18^ Two MRC randomized trials reported 54%‐84% symptomatic improvement in hemoptysis after palliative thoracic RT.^25^, ^26^ Retrospective studies of palliative gastric RT reported response rates of up to 74% for bleeding, 67% for pain and 68% for obstruction.^19^ However, the interpretation of the results from these trials are limited by the poor definition of endpoints and limited reporting of treatment toxicities. Other limitations included small sample size, use of 2‐dimensional RT techniques and wide range of dose fractionation regimens.^27^, ^28^ In addition, there is currently no available data on HRQOL outcomes for palliative gastric RT. HRQOL outcomes should be included in prospective studies of palliative RT as they allow measurement and comparison of the effects of palliative treatment. All patients in the current study were treated with 3‐dimensional conformal RT with anterior‐posterior and lateral fields which might have accounted for the low rates of treatment toxicity.

There is currently no standard fractionation regimen for palliative gastric RT. A systematic review suggested that low BED regimens are as effective as high BED regimens for palliation of bleeding.^19^ However, while there may be no difference between low and high BED regimens in symptom palliation, local control may be improved with higher BED regimens. One study showed a trend for poorer local control with low BED regimens of ≤39 Gy compared with higher BED regimens.^16^ Similarly, Kim et al from MD Anderson also suggested that low BED regimens of <41 Gy predicted for poorer local control compared to higher BED regimens.^20^

The strengths of our study include the use of a standardized radiation treatment protocol with well‐defined endpoints and HRQOL assessments. The limitations of our study are that this study relied on historical controls for estimation of expected response rates. This has been shown to be associated with fairly high false positive rates. However, we acknowledge and accept this compromise given that there are no prior prospective studies to base our sample size calculation on. Although this trial did not meet its prespecified sample size due to slow accrual, the current sample size of 50 patients will give us 90% statistical power with the same sample size calculation assumptions.

In conclusion, this study shows that palliative gastric RT is effective and well tolerated and resulted in improvement in fatigue, dysphagia and pain at the end of RT and at 1 month after the completion of RT in a significant proportion of patients. A phase III study comparing this fractionation with a shorter fractionation regimen is planned.

## CONFLICT OF INTEREST

The authors declare no conflict of interest.
